# Characterization of the cytolethal distending toxin (typhoid toxin) in non-typhoidal *Salmonella* serovars

**DOI:** 10.1186/s13099-015-0065-1

**Published:** 2015-07-24

**Authors:** Lorraine D Rodriguez-Rivera, Barbara M Bowen, Henk C den Bakker, Gerald E Duhamel, Martin Wiedmann

**Affiliations:** Department of Food Science, College of Agriculture and Life Sciences, Cornell University, 347 Stocking Hall, Ithaca, NY 14853 USA; Department of Biomedical Sciences, College of Veterinary Medicine, Cornell University, Ithaca, NY 14853 USA; Department of Veterinary Integrative Biosciences, College of Veterinary Medicine and Biomedical Sciences, Texas A&M University, College Station, TX 77843-4458 USA; Department of Animal and Food Sciences, College of Agricultural Sciences and Natural Resources, Lubbock, TX 79409 USA

**Keywords:** *Salmonella*, Non-typhoidal, Typhi, Toxin, Typhoid toxin, CdtB, PltA, PltB

## Abstract

**Background:**

For many putative *Salmonella enterica* subsp. *enterica* virulence genes, functional characterization across serovars has been limited. Cytolethal distending toxin B (CdtB) is an incompletely characterized virulence factor that is found not only in *Salmonella enterica* subsp. *enterica* serovar Typhi (*Salmonella* Typhi) and dozens of Gram negative bacterial pathogens, but also in non-typhoidal *Salmonella* (NTS) serovars.

**Methods:**

A comparative genomics approach was performed to characterize sequence conservation of the typhoid toxin (TT), encoded in the CdtB-islet, between *Salmonella* Typhi and NTS serovars. The cytotoxic activity of representative *Salmonella enterica* subsp. *enterica* serovars Javiana, Montevideo and Schwarzengrund strains and their respective isogenic *cdtB* mutants was determined in human intestinal epithelial Henle-407 cells by assessment of cell cycle progression of infected cells using fluorescence-activated cell sorting (FACS). Two-way analysis of variance (ANOVA) was used to determine whether *cdtB* deletion had a significant (p < 0.05) effect on the percentage of Henle-407 cells at each stage of the cell cycle.

**Results:**

Here we show that a CdtB-islet encoding the cytolethal distending toxin B (CdtB), pertussis-like toxin A (PltA), and pertussis-like toxin B (PltB) is present in a dozen NTS serovars and that these proteins have a high level of sequence conservation and each form monophyletic clades with corresponding *Salmonella* Typhi genes. Human epithelial Henle-407 cells infected with three representative CdtB-encoding NTS serovars displayed G_2_/M phase cell cycle arrest that was absent in cells infected with corresponding isogenic *cdtB* null mutants (p < 0.0001 for the factor ∆*cdtB* deletion).

**Conclusion:**

Our results show that CdtB encoded by NTS serovars has a genomic organization, amino acid sequence conservation and biological activity similar to the TT, and thus, may contribute to disease pathogenesis.

**Electronic supplementary material:**

The online version of this article (doi:10.1186/s13099-015-0065-1) contains supplementary material, which is available to authorized users.

## Background

The cytolethal distending toxin B (CdtB) is a recently recognized virulence factor of *Salmonella enterica* subsp. *enterica* serovar Typhi [1], as well as a wide range of other Gram negative bacterial pathogens in the *Gamma* and *Epsilon* classes of *Proteobacteria* [2]. Host cells intoxicated with CdtB display a DNA damage response (DDR) characterized by irreversible cell cycle arrest, with persistent DDR leading to cell death by apoptosis [2]. Multi-locus sequence analysis data reported by our group have previously shown an unambiguous subdivision of *S. enterica* subsp. *enterica* into at least two populations that we designated clades A and B [3]; this subdivision has been confirmed by other studies [4, 5]. Clades A and B not only represents well supported subdivisions, but also show distinct genomic features that strongly suggests niche specialization of these subpopulations; for example clade B strains contain several clade specific genes or operons, including a ß-glucuronidase operon, a S-fimbrial operon, and clade B specific cell surface related genes [3]. The study by den Bakker et al. [3] also identified an islet encoding the cytolethal distending toxin B (CdtB-islet) in the genomes of 56 non-typhoidal *Salmonella* isolates, including (i) 37/38 clade B isolates, 14/115 non-typhoidal clade A isolates, and five isolates that did not clearly group into clade A or B [3]. Despite these observations and the important role of non-typhoidal *Salmonella* as foodborne and zoonotic pathogens, the role of CdtB in non-typhoidal serovars has remained understudied; studies that explore the functionality of CdtB in non-typhoidal serovars are thus essential.

In *Salmonella* Typhi, the CdtB-islet includes five genes, namely *pltA*, *pltB*, *ttsA*, *sty1887*, in addition to *cdtB*. *pltA* and *pltB* encode homologs of pertussis toxin components, which are responsible for ADP-ribosylation of a host protein [6] and export of CdtB from the *Salmonella* containing vacuole as well as from infected host cells. CdtB, PltA, and PltB are the three subunits that form the so called “typhoid toxin” in *Salmonella* Typhi. *ttsA* encodes a phage-origin muramidase necessary for the secretion of the PltA/CdtB/PltB toxin [7]. *sty1887* encodes a putative homolog of a phage tail protein; deletion of this gene in *Salmonella* Typhi did not affect secretion of CdtB [7]. While other Gram-negative bacteria also contain *cdtB*, these other species typically do not contain homologs of *pltA* and *pltB*. In these other Gram-negative species toxin import into host cell compartments and into the cytosol appears to typically be facilitated by CdtA and CdtC; in these bacteria CdtA, CdtB and CdtC are protein subunits and assemble into a single holotoxin [2]. Importantly, CdtA and CdtC show no homologies with PltA and PltB.

On the basis of the well-documented role of CdtB in host-pathogen interactions in *Salmonella* Typhi and other bacterial pathogens, we hypothesized that CdtB may also play a critical role in host cell interactions of non-typhoidal *Salmonella* serovars newly identified as encoding CdtB. We used a comparative genomics approach to characterize sequence conservation of *pltA*, *pltB*, and *cdtB* among *S.* Typhi and non-typhoidal *Salmonella* serovars. To confirm the biological activity of CdtB in non-typhoidal *Salmonella* serovars, we created *cdtB* null mutants in three representative non-typhoidal *Salmonella* strains and assessed the cell cycle of infected Henle-407 human epithelial cells.

## Results

### Phylogenetic analysis revealed that CdtB encoded in the genomes of NTS serovars has a high level of sequence conservation when compared to *S.* Typhi’s CdtB

**Fig. 1:**
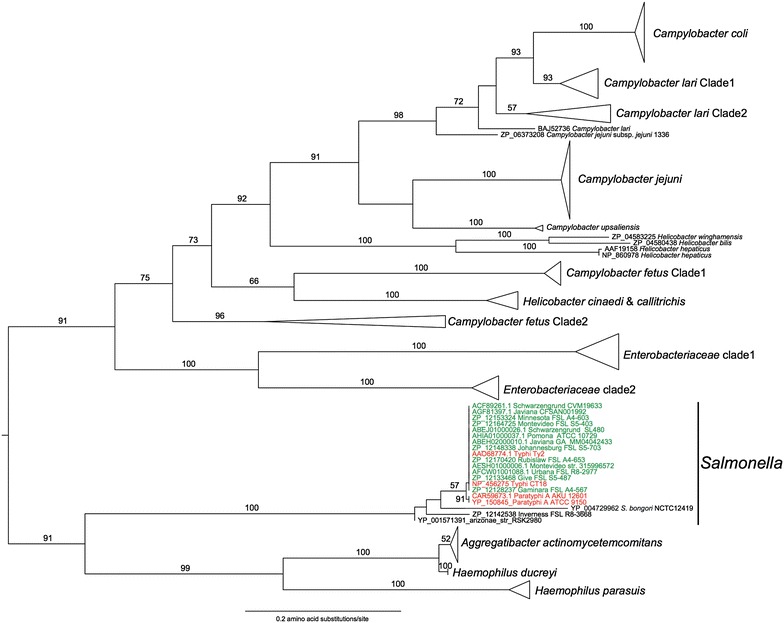
Amino acid based maximum likelihood phylogeny of CdtB. Non-typhoidal *Salmonella enterica* subsp. *enterica* serovars are colored *green*, while *S.* Typhi and Paratyphi A accessions are colored *red*. Values on or next to the branches are bootstrap values based on 250 bootstrap replicates.

A maximum likelihood (ML) based phylogeny of CdtB amino acid sequences showed that homologs of this gene are widely distributed among Gram-negative bacteria (Fig. 1), including 11 serovars classified into *S. enterica* subsp. *enterica* clade B (13 isolates, Fig. 1), *S. enterica* subsp. *enterica* serovar Inverness (FSL R8-3668), *S. enterica* subsp. *arizonae* (RSK2980), and *S. bongori* (NCTC 12419). All *S. enterica* genomes that contained CdtB also encoded PltA and PltB, these genes are characteristic of the CdtB-islet, which encodes the components of the *S.* Typhi typhoid holotoxin complex [8]. In 18/20 *cdtB* positive *S. enterica* strains, we also identified paralogs of *pltA* and *pltB* in a genomic region that was not within the CdtB-islet. These paralogs have previously been annotated as the *artA/artB* operon in *S*. Typhimurium DT104 [9] and encode an AB5 toxin [10]. *S.* Typhi CdtB, PltA, and PltB show high levels of homology with the corresponding proteins in non-typhoidal *S. enterica* subsp. *enterica* isolates (with the exception of *S.* Inverness) with 99.3–100% amino acid (aa) sequence identity for CdtB (Fig. 1), 98.3–100% aa sequence identity for PltA (Additional file 1), and 94.9–100% aa sequence identity for PltB (Additional file 2). The high level of sequence conservation for CdtB is further confirmed by the high (>98%) aa sequence identity among the 12 *S. enterica* subsp. *enterica* serovars (including Typhi and Paratyphi A) (Fig. 1). Moreover, functionally critical residues (i.e., PltA Cys 214, CdtB Cys 269) within the typhoid holotoxin proteins, as determined by Song et al. [8], were conserved in all 14 non-typhoidal *Salmonella* genomes examined (Additional file 3).

### Henle-407 human epithelial cells infected with typhoid toxin-producing NTS strains displayed cell arrest in G_2_/M phase of the cell cycle

**Fig. 2:**
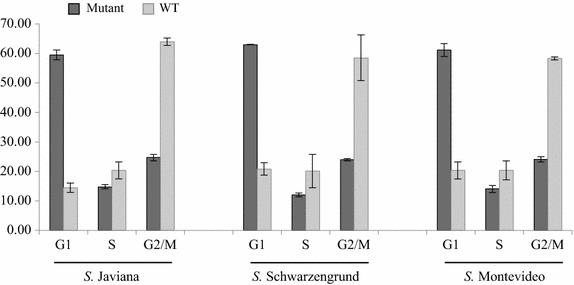
Cell cycle analysis of human epithelial Henle-407 cells infected with non-typhoidal *Salmonella* wild type (WT) strains and their corresponding isogenic *cdtB* mutants. Cells infected with *Salmonella* WT strains and their isogenic mutants were analyzed by FACS at 72 h of post-infection. Data shown represent the averages of two independent biological replicates with two technical replicates (two infected wells per experiment) each. Y axis indicates percentage of cells in G_1_, S, and G_2_/M phase (as indicated on the X axis); the bars indicate the range of values. Cells infected with *Salmonella* WT parent strains (i.e., *S*. Montevideo, *S*. Schwarzengrund, and *S*. Javiana) showed significantly higher percentage cells in G_2_/M phase compared to the corresponding isogenic ∆*cdtB* mutants (p < 0.0001 for the factor ∆*cdtB* deletion; ANOVA). Conversely, cells infected with the isogenic ∆*cdtB* mutants showed significantly higher percent cells in G_1_ phase (p < 0.0001; ANOVA) compared to the corresponding parent WT strains.

Given the high level of sequence conservation for all three typhoid toxin subunits (i.e., CdtB, PltA, PltB) including conservation of functionally critical aa residues, we hypothesized that a functional typhoid holotoxin is produced by non-typhoidal salmonellae. Therefore, we constructed isogenic mutants with deletions of *cdtB* in three non-typhoidal *Salmonella enterica* strains, classified in clade B, for further phenotypic characterization. One representative strain of *Salmonella* serovars Javiana, Montevideo, and Schwarzengrund was selected because these serovars have been responsible for several large outbreaks in the last 10 years [11–14] in the United States. In addition, *S.* Javiana and *S.* Montevideo are among the six most frequently reported serovars isolated from humans in the US [15]. When Henle-407 cells were infected with parent strains and corresponding isogenic mutants, a clear cell cycle arrest was observed at 72 h post-inoculation, using a fluorescence-activated cell sorter (FACS). Specifically, Henle-407 cells infected with *Salmonella* parent strains were arrested in the G_2_/M phase of the cell cycle (Fig. 2); 60.3% of Henle cells infected with the parent strains were in the G_2_/M phase (average across all three serovars), which is significantly higher as compared to the Henle cells infected with the *cdtB* mutant strains (24.3% average across all three serovars; p < 0.0001; see Fig. 2). By contrast, 18.6% of Henle cells infected with the parent strains (average across all three serovars) were in G_1_ phase, which is significantly (p < 0.0001; Fig. 2) lower as compared to Henle cells infected with the corresponding *SalmonellacdtB* null mutants (61.2% average across all three serovars) and compared to uninfected cells (60.0%). Serovar did not have a significant effect, and there were no significant interactions between *cdtB* status (mutant vs. wild type) and serovar. These findings show that non-typhoidal *Salmonella* serovars produce a functional typhoid toxin that causes G_2_/M cell cycle arrest in human epithelial cells. While some may argue that complementation of the *cdtB* mutants would be needed to confirm these findings, the fact that we found consistent phenotypes across three distinct serovars provides strong evidence that the effects shown are due to the mutations introduced. Although *S.* Typhi was not included as a control in our experiments, the patterns of cell cycle arrest were similar to those previously reported; for example, Spanò et al. [6] showed that 84% of Typhi infected Henle cells were in G_2_/M phase as compared to 16% of cells infected with Typhi Δ*cdtB* and 14% of untreated cells.

### *Salmonella* serovars that showed a higher proportion of cases with invasive disease were significantly (*p* < 0.05) more likely to be *cdtB* positive

To determine whether *cdtB* positive serovars are more likely to be associated with invasive disease, we utilized previously reported epidemiological data on association of disease severity with different *Salmonella* serovars; this study used data for 46,639 human cases reported in the US between 1996 and 2006 [16]. Serovars were classified as *cdtB* positive if all isolates of a given serovar characterized by den Bakker et al. [3] were reported to be positive for *cdtB*. Among the serovars in this study, 13 showed a significantly higher proportion of invasive disease cases (as compared to *Salmonella* Typhimurium), while 35 showed a lower or equal proportion of invasive disease cases. When serovars were classified as *cdtB* positive or negative, based on data reported by den Bakker et al. [3], 8 of the 13 serovars that showed a higher proportion of invasive disease cases could be classified as *cdtB* positive serovars (i.e., Oranienburg, Poona, Schwarzengrund, Panama, Sandiego, Brandenburg, Muenster, Urbana). Among the 35 serovars that showed an equal or lower proportion of invasive disease cases, only eight were classified as *cdtB* positive (i.e., Javiana, Miami, Montevideo, Rubislaw, Gaminara, Kiambu, Johannesburg and Give). A Fisher’s exact test showed that serovars that showed a higher proportion of cases with invasive disease were significantly (p < 0.05) more likely to be *cdtB* positive as compared to serovars that showed a lower proportion of cases with invasive disease.

## Discussion

A comparative genomic study by den Bakker et al. [3] previously revealed the presence of a CdtB-islet within the genomes of 56 non-typhoidal *Salmonella* isolates, of which approximately 70% belonged to a restricted subpopulation of *S. enterica* subsp. *enterica* (clade B). Subsequently, Desai et al. [17] reported the presence of *cdtB* (referred to as typhoid toxin in that study) in the genomes of 2 *S. enterica* subsp. *diarizoniae* and 2 *S. enterica* subsp. *arizoniae* strains. Consistent with previous analyses [18], the present comparative genomic analyses confirmed that homologs of *Salmonella* Typhi *cdtB* are widely distributed among Gram-negative bacterial pathogens. Contribution of CdtB to disease pathogenesis has been reported in dozens of bacterial pathogens, including reduced cytotoxicity of *cdtB* null mutants (e.g., *H. ducreyi*, *H. hepaticus*, *C. jejuni*) and CdtB mediated G_2_/M cell cycle arrest (e.g., *E. coli*, *A. actinomycetemcomitans*, *H. ducreyi, H. hepaticus*) [2, 19]. Importantly, our sequence analysis shows that the genes encoded by the CdtB-islet of *S*. Typhi and non-typhoidal serovars are highly conserved and share a common phylogenetic ancestor, providing new evidence of a functional CdtB in a large subset of pathogenic non-typhoidal *Salmonella*.

Phenotypic characterization of *cdtB* null mutants in three different serovar backgrounds confirmed that CdtB is functional across different non-typhoidal *Salmonella* and required for induction of G_2_/M phase cell cycle arrest of intoxicated human intestinal epithelial Henle-407 cells. While our findings are consistent with previous reports that *S*. Typhi CdtB is directly responsible for G_2_/M phase cell cycle arrest in infected eukaryotic host cells [1, 6–8], our data provide important new evidence that indicate a contribution of CdtB in disease pathogenesis across a large number of human non-typhoidal *Salmonella* strains. Importantly CdtB-islet positive strains may also cause cell cycle arrest in the infected target tissues, which may increase the risk of long-term sequelae. This is consistent with Lara-Tejero & Galán [20] who suggested that exposure to CdtB and the associated DDR may predispose individuals infected with *C. jejuni* to intestinal cancer. Consistent with our results, a recent study also confirmed the presence of a functional CdtB in a strain of *Salmonella* Javiana [21]; this study also showed that the cell cycle arrest pattern observed in cells infected with the *Salmonella* Typhi control strain was similar to that observed for the *Salmonella* Javiana test strain. Another recent study also showed that a *Salmonella* Javiana *cdtB* mutant strain did not adhere more to mice macrophages when compared to the wild type (WT) [22]. Previous studies with *Salmonella* Typhi [1] have also demonstrated that CdtB is only synthesized when *Salmonella* is within the intracellular compartment. As IgeR, which has been found to repress *Salmonella* Typhi *cdtB* transcription in extracellular environments by binding to the *cdtB* promoter, is conserved across typhoidal and non-typhoidal *Salmonella* serovars, IgeR may also downregulates *cdtB* transcription in extracellular environments in non-typhoidal serovars. Future studies are needed though to evaluate regulation of CdtB expression in non-typhoidal serovars in order further understand the specific role of CdtB in non-typhoidal *Salmonella* serovars.

## Conclusion

Our findings highlight the possibility that non-typhoidal *Salmonella* may represent distinct CdtB-islet positive and negative subgroups, which differ in pathogenetic mechanisms and host interactions. Further experimental work, including characterization of *cdtB* mutants in animal models, is clearly needed though to more completely define the contributions of CdtB and the other proteins encoded in the CdtB-islet, as well as the *artAB* operon, to pathogenesis of a growing list of non-typhoidal *Salmonella* serovars recognized as encoding these virulence factors; our mutants are freely available to other researchers for these types of experiments. Given the association of several CdtB-producing bacterial pathogens with cancer [23, 24] and the ability of CdtB to cause a DDR in a wide range of eukaryotic cells, it would also be important to explore whether CdtB-producing bacteria can promote cancer development in their respective hosts. As an analysis of previously reported data on distribution of invasive and non-invasive disease among patients infected with different *Salmonella* serovars provided preliminary support that *cdtB* positive isolates may be more likely to cause invasive disease, future epidemiological studies are also needed to determine whether *cdtB*-positive *Salmonella* isolates are associated with different or more severe disease outcomes in human hosts, in addition to animal studies on the role of CdtB in invasive infection by non-typhoidal *Salmonella*.

## Methods

### PltA, PltB, and CdtB phylogeny and amino acid sequence analysis

PltA, PltB and CdtB amino acid sequences of *S.* Typhi CT18 were obtained from GenBank. *Salmonella* genomes (both finished and draft, obtained from the nr/nt and WGS databases at NCBI) were queried for homologs of *S*. Typhi CT18 PltA, PltB and CdtB using both nucleotide and protein blast searches (blastp and blastn, respectively). Nucleotide sequences obtained from these searches were translated into amino acid sequences and aligned using MAFFT [25]. Maximum likelihood phylogenies were created with PhyML (version 20130708), using the WAG model of amino acid substitution and a gamma distribution of variable sites. To assess the robustness of the inferred phylogeny 250 bootstrap replicates were performed for each analysis.

### Bacterial strains

Three non-typhoidal *Salmonella* strains representing serovars Javiana (isolate FSL S5-0395), Schwarzengrund (FSL R6-0879), and Montevideo (FSL R8-4841) were obtained from the New York State Department of Health; all strains were from humans with clinical symptoms of salmonellosis. The presence of *plt*A, *plt*B, and *cdt*B in these strains was confirmed by TaqMan^®^ assays (Life Technologies) as previously described [3]. Isogenic ∆*cdtB* mutants were constructed by using the Lambda Red system as previously described [26] and *cdtB* deletions were confirmed by PCR and sequencing of the deletion allele (see Additional file 4).

### Henle-407 cell infection

Human epithelial Henle-407 cells (ATCC CCL-6) were grown at 37°C in 5.0% CO_2_ atmosphere and Dulbecco’s Modified Eagle Medium (DMEM; Corning) supplemented with 10% fetal bovine serum (FBS; Atlanta Biologicals). For infection studies, bacterial cultures were prepared as previously described [1], with some modifications; all *Salmonella* growth steps were performed in Lysogeny Broth (LB, commonly referred to as Luria–Bertani broth) with 0.3 M NaCl at 37°C, without shaking. Briefly, overnight *Salmonella* cultures were diluted 1:100 in fresh LB-NaCl broth and incubated at 37°C until they reached OD_600_ of 0.4. Then, these cultures were diluted 1:100 into Nephelo culture flasks with 50 mL LB-NaCl broth, and incubated at 37°C until the cultures reached an OD_600_ of 0.4, followed by incubation for an additional 3 h to yield a final density of approx. 1 × 10^9^ CFU/mL. Infection studies were performed with Henle-407 cells seeded in 6-well plates and incubated for 24 h before inoculation; media was replaced with fresh DMEM-FBS 30 min before inoculation with *Salmonella* at an MOI of 50. After incubation at 37°C and 5.0% CO_2_ for 1 h, the cells were washed with phosphate buffered saline (PBS) followed by incubation in fresh DMEM-FBS containing gentamicin (100 μg/mL) for 1 h. After 1 h, the Henle-407 cells were washed 3 times with PBS and fresh DMEM-FBS with gentamicin (10 μg/mL) was added, followed by incubation for another 72 h. At the end of the incubation period, the uninfected control and infected Henle-407 cells were processed for cell cycle analysis with a fluorescence-activated cell sorter (FACS), as described below. All assays were performed in duplicate in two separate biological replicates with each *Salmonella* strain.

### Cell cycle analysis

The cell cycle of uninfected control and infected Henle-407 cells was determined by FACS analysis as described previously [20], with some modifications. Briefly, Henle-407 cells were washed, trypsinized, and centrifuged at 1,500 rpm for 5 min at room temperature. The supernatant was removed, the cells were fixed by adding cold 70% ethanol (while vortexing at slow speed) and kept at –20°C for at least 1 h before adding PBS containing 0.1% (v/v) Tween 20 and 1% (w/v) BSA (PBST). Then the cells were incubated for 10 min at room temperature, washed three times in PBST before re-suspending in propidium iodide (PI; Sigma-Aldrich) staining solution [40 μg of PI/mL, 100 μg of RNase A/mL (Sigma-Aldrich)], followed by incubation at room temperature in the dark for an additional 10 min. Subsequent DNA content analysis of approximately 3 × 10^4^ cells was performed with a LSRII Flow Cytometer (BD-Biosciences). The percentages of Henle-407 cells in G_1_, S, and G_2_/M phase of the cell cycle were calculated after quantifying the mean percentages of the cells detected in manually adjusted gates for 2N, 3N, and 4N DNA contents.

### Statistical analysis

Data were imported into a commercially available statistical software program (SAS, version 9.2; SAS Institute Inc., Cary, NC, USA) for analysis. Two-way analysis of variance (ANOVA) was used to determine whether *cdtB* deletion had a significant (p < 0.05) effect on the percentage of Henle-407 cells at the G_1_, S, and G_2_/M stages of the cell cycle; each measurement represented the mean of two technical replications. CdtB status (mutant vs. wild type) and serovar were included as factors in the analysis, and their interaction was also evaluated. Tukey’s test was used to investigate any differences in the means.

## Additional files

Additional file 1: Amino acid based maximum likelihood phylogeny of PltA. Non-typhoidal *Salmonella* *enterica* subsp. *enterica* serovars are colored green, *S*. Paratyphi A accessions are colored orange, and *S*. Typhi accessions are colored red. Values on or next to the branches are bootstrap values based on 250 bootstrap replicates. Additional file 2: Amino acid based maximum likelihood phylogeny of PltB. Non-typhoidal *Salmonella* *enterica* subsp. *enterica* serovars are colored green, *S*. Paratyphi A accessions are colored orange, and *S*. Typhi accessions are colored red. Values on or next to the branches are bootstrap values based on 250 bootstrap replicates. Additional file 3:
*Salmonella* genomes and sequences used for *cdtB*, *pltA*, and *pltB* analyses. Table in a Microsoft excel file listing the accession numbers of the genomes used for phylogenetic analysis. Additional file 4: Sequence of *cdtB* and surrounding region, indicating that deletion is internal to *cdtB*; *cdtB* start and stop codons are boxed. Primer sequences in bold. Primer sequence homologous to pKD4 template primer P1 and P2 are italicized. ‘Scar’ sequence from pKD4 in orange. The sequence shown is for the serovar Javiana mutant; the serovar Schwarzengrund and Montevideo mutants showed equivalent sequences. (B) Organization of the region that contains *pltB*, *pltA*, and *cdtB*, which indicates that the *cdtB* deletion shown in (a) will not disrupt *pltA* or *pltB*. (C) Gel that shows the PCR product for the *cdtB* deletion alleles in serovars Schwarzengrund (lane 1), Montevideo (lane 4), and Javiana (lane 7); these PCR products were used to generate the sequencing data shown in part (A).
