# Supplementary figures and images for: Characterization of the cytolethal distending toxin (typhoid toxin) in non-typhoidal Salmonella serovars
Source: Gut Pathog. 2015 Jul 24;7:19. doi: 10.1186/s13099-015-0065-1 (PMC4511993; doi:10.1186/s13099-015-0065-1)

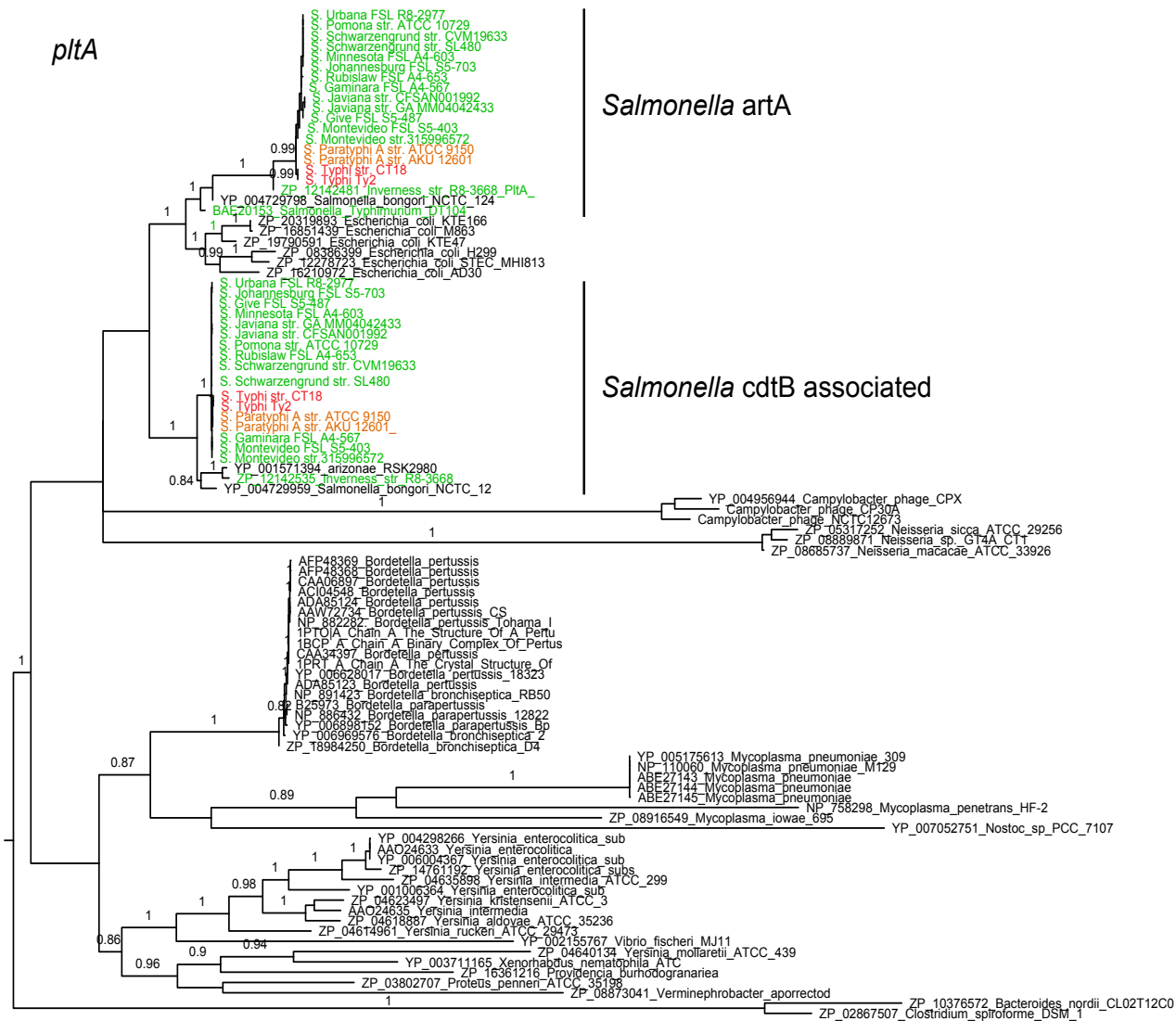

2.0 Amino acid substitutions/site

Supplement: Additional file 1: — Amino acid based maximum likelihood phylogeny of PltA. Non-typhoidal Salmonella enterica subsp. enterica serovars are colored green, S. Paratyphi A accessions are colored orange, and S. Typhi accessions are colored red. Values on or next to the branches are bootstrap values based on 250 bootstrap replicates. [file 13099_2015_65_MOESM1_ESM.pdf]
