# Supplementary material for: Characterization of the cytolethal distending toxin (typhoid toxin) in non-typhoidal Salmonella serovars
Source: Gut Pathog. 2015 Jul 24;7:19. doi: 10.1186/s13099-015-0065-1 (PMC4511993; doi:10.1186/s13099-015-0065-1)
Supplement: Additional file 2: — Amino acid based maximum likelihood phylogeny of PltB. Non-typhoidal Salmonella enterica subsp. enterica serovars are colored green, S. Paratyphi A accessions are colored orange, and S. Typhi accessions are colored red. Values on or next to the branches are bootstrap values based on 250 bootstrap replicates. [file 13099_2015_65_MOESM2_ESM.pdf]

pltB

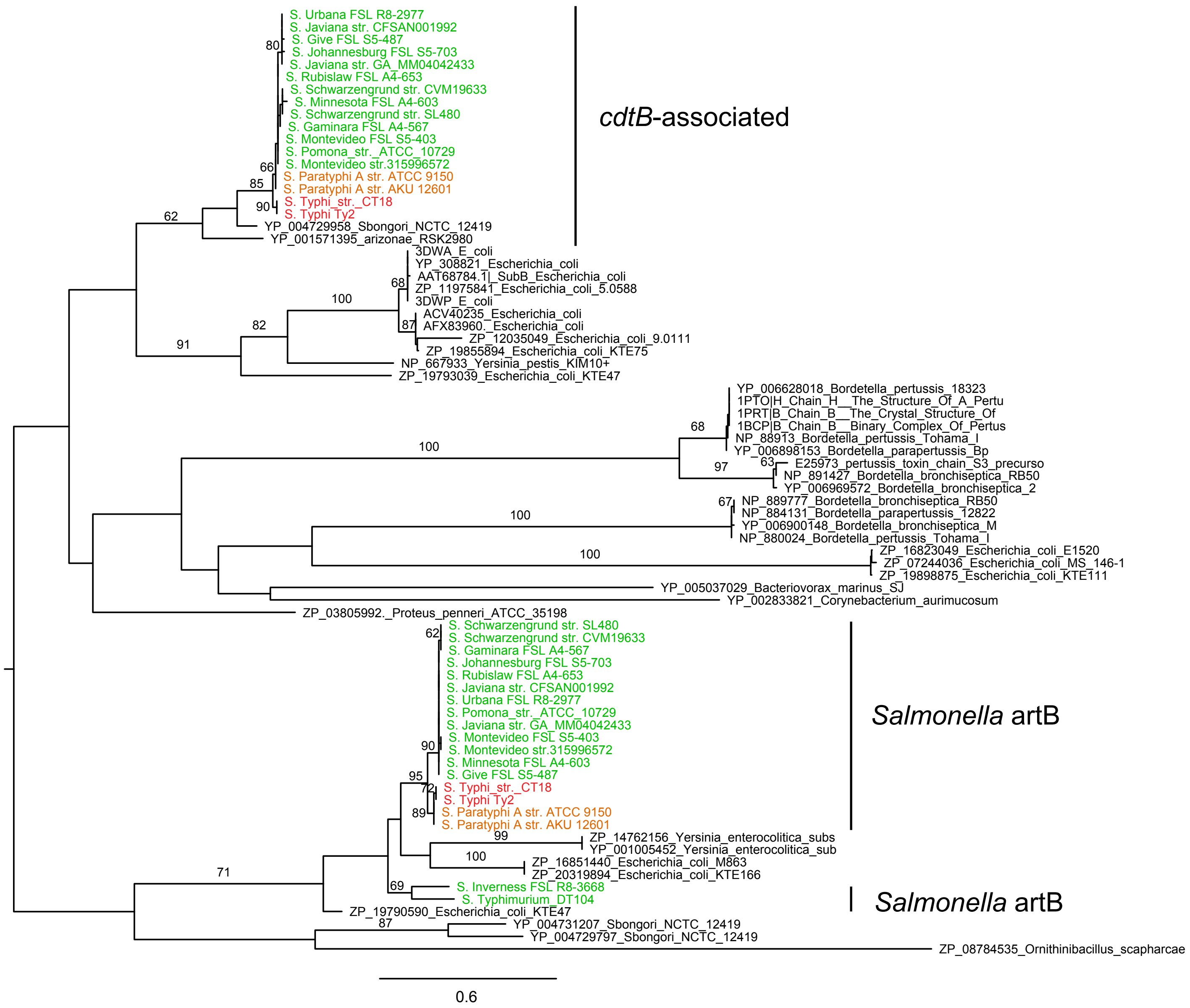
S. Urbana FSL R8-2977
S. Javiana str. CFSAN001992
S. Give FSL S5-487
S. Johannesburg FSL S5-703
S. Javiana str. GA_MM04042433
S. Rubislaw FSL A4-653
S. Schwarzengrund str. CVM19633
S. Minnesota FSL A4-603
S. Schwarzengrund str. SL480
S. Gaminara FSL A4-567
S. Montevideo FSL S5-403
S. Pomona_str._ATCC_10729
S. Montevideo str.315996572
S. Paratyphi A str. ATCC 9150
S. Paratyphi A str. AKU 12601
S. Typhi_str._CT18
S. Typhi Ty2
YP_004729958_Sbongori_NCTC_12419
YP_001571395_arizonae_RSK2980
cdtB-associated
3DWA_E_coli
YP_308821_Escherichia_coli
AAT68784.1|_SubB_Escherichia_coli
ZP_11975841_Escherichia_coli_5.0588
3DWP_E_coli
ACV40235_Escherichia_coli
AFX83960._Escherichia_coli
ZP_12035049_Escherichia_coli_9.0111
ZP_19855894_Escherichia_coli_KTE75
NP_667933_Yersinia_pestis_KIM10+
ZP_19793039_Escherichia_coli_KTE47
YP_006628018_Bordetella_pertussis_18323
1PTO|H_Chain_H__The_Structure_Of_A_Pertu
1PRT|B_Chain_B__The_Crystal_Structure_Of
1BCP|B_Chain_B__Binary_Complex_Of_Pertus
NP_88913_Bordetella_pertussis_Tohama_I
YP_006898153_Bordetella_parapertussis_Bp
E25973_pertussis_toxin_chain_S3_precurso
NP_891427_Bordetella_bronchiseptica_RB50
YP_006969572_Bordetella_bronchiseptica_2
NP_889777_Bordetella_bronchiseptica_RB50
NP_884131_Bordetella_parapertussis_12822
YP_006900148_Bordetella_bronchiseptica_M
NP_880024_Bordetella_pertussis_Tohama_I
ZP_16823049_Escherichia_coli_E1520
ZP_07244036_Escherichia_coli_MS_146-1
ZP_19898875_Escherichia_coli_KTE111
YP_005037029_Bacteriovorax_marinus_SJ
YP_002833821_Corynebacterium_aurimucosum
ZP_03805992._Proteus_penneri_ATCC_35198
S. Schwarzengrund str. SL480
S. Schwarzengrund str. CVM19633
S. Gaminara FSL A4-567
S. Johannesburg FSL S5-703
S. Rubislaw FSL A4-653
S. Javiana str. CFSAN001992
S. Urbana FSL R8-2977
S. Pomona_str._ATCC_10729
S. Javiana str. GA_MM04042433
S. Montevideo FSL S5-403
S. Montevideo str.315996572
S. Minnesota FSL A4-603
S. Give FSL S5-487
S. Typhi_str._CT18
S. Typhi Ty2
S. Paratyphi A str. ATCC 9150
S. Paratyphi A str. AKU 12601
Salmonella artB
ZP_14762156_Yersinia_enterocolitica_subs
YP_001005452_Yersinia_enterocolitica_sub
ZP_16851440_Escherichia_coli_M863
ZP_20319894_Escherichia_coli_KTE166
S. Inverness FSL R8-3668
S. Typhimurium_DT104
Salmonella artB
ZP_19790590_Escherichia_coli_KTE47
YP_004731207_Sbongori_NCTC_12419
YP_004729797_Sbongori_NCTC_12419
ZP_08784535_Ornithinibacillus_scapharcae
80
66
85
90
62
68
100
87
82
91
68
100
97
63
67
100
100
62
90
95
72
89
99
100
71
69
87
0.6
