# Supplementary material for: Characterization of the cytolethal distending toxin (typhoid toxin) in non-typhoidal Salmonella serovars
Source: Gut Pathog. 2015 Jul 24;7:19. doi: 10.1186/s13099-015-0065-1 (PMC4511993; doi:10.1186/s13099-015-0065-1)
Supplement: Additional file 4: — Sequence of cdtB and surrounding region, indicating that deletion is internal to cdtB; cdtB start and stop codons are boxed. Primer sequences in bold. Primer sequence homologous to pKD4 template primer P1 and P2 are italicized. ‘Scar’ sequence from pKD4 in orange. The sequence shown is for the serovar Javiana mutant; the serovar Schwarzengrund and Montevideo mutants showed equivalent sequences. (B) Organization of the region that contains pltB, pltA, and cdtB, which indicates that the cdtB deletion shown in (a) will not disrupt pltA or pltB. (C) Gel that shows the PCR product for the cdtB deletion alleles in serovars Schwarzengrund (lane 1), Montevideo (lane 4), and Javiana (lane 7); these PCR products were used to generate the sequencing data shown in part (A). [file 13099_2015_65_MOESM4_ESM.pdf]

A

Parent   TTATTGACGTTAGACTATGTATCTTACTATCTGCACAAGACATAGAACCAGAAACAAGTCAGGCATTGCCA
Mutant   TTATTGACGTTAGACTATGTATCTTACTATCTGCACAAGACATAGAACCAGAAACAAGT**CAGGCATTGCCA**

Parent   GAATAAAATCAACGTCATGAAACA**ATG**AAAAAACCTGTTTTTTTCCTTCTGACCATGATCATCTGCAGCTA
Mutant   **GAATAAAATCAACGTCATGAAACA*GTGTAGGCTGGAGCTGCTTC***GAAGTTCCTATACTTTCTAGAGAATAG

Parent   TATTTCTTTTGCCTGCGCTAATATCAGTGACTACAAAGTTATGACCTGGAATCTTCAGGGCTCTTCAGCAT
Mutant   GAACTTCGGAATAGGAA--

Parent   CTACAGAAAGTAAATGGAATGTCAATGTCAGACAGCTTTTAAGTGGTACTGCCGGTGTGGATATTCTTA
Mutant   ---

Parent   TGGTACAGGAGGCCGGTGCTGTTCCCACCTCAGCGGTTCCTACCGGACGACATATTCAGCCTTTTGGAGT
Mutant   --

Parent   GGGTATTCCCATTGATGAATACACCTGGAATCTCGGAACCACCAGCCGTCAGGATATAAGATATATCTAC
Mutant   --

Parent   CACTCGGCTATTGATGTTGGAGCACGCCGTGTTAATCTGGCAATAGTTTCCAGACAAAGAGCGGATAATG
Mutant   --

Parent   TTTATGTCTTGCGTCCGACAACTGTCGCATCTCGCCCCGTCATTGGCATCGGACTGGGTAATGATGTTTTTC
Mutant   --

Parent   TGACAGCGCACGCACTGGCTAGTGGAGGTCCGGATGCTGCAGCTATTGTCAGGGTTACCATTAATTTTTT
Mutant   --

Parent   TAGACAACCTCAGATGCGGCATTTATCCTGGTTTCTTGCCGGGGATTTTAATCGCAGCCCAGACAGACTTG
Mutant   ---

Parent   AAAATGACCTGATGACTGAGCATCTGGAACGAGTTGTAGCCGTACTCGCACCTACAGAACCCACGCAAATT
Mutant ---

Parent   GGCGGTGGTATTTTAGATTATGGGGTCATTGTCGATCGAGCACCTTATTCACAAAGGGTTGAAGCTCGCTT
Mutant ---

Parent   CTGATCATTATCCCGTAGCCTTTTTGGCACGAAGCTGT**TAATGAGTAAATAGCGTTGACGACATGTACTTTG**
Mutant   -------------------***CTAAGGAGGATATTCATATGGACC*TGAGTAAATAGCGTTGACGACATGTACTTTG**

Parent   **AGCGTAAGGTGCAGA**ATATATATTTATTATAAGGTAAAATTATGAGTTGTTTTACCAGTCCAGCAATTAT
Mutant   **AGCGTAAGGTGCAGA**ATATATATTTATTATAAGGTAAAATTATGAGTTGTTTTACCAGTCCAGCAATTAT

B

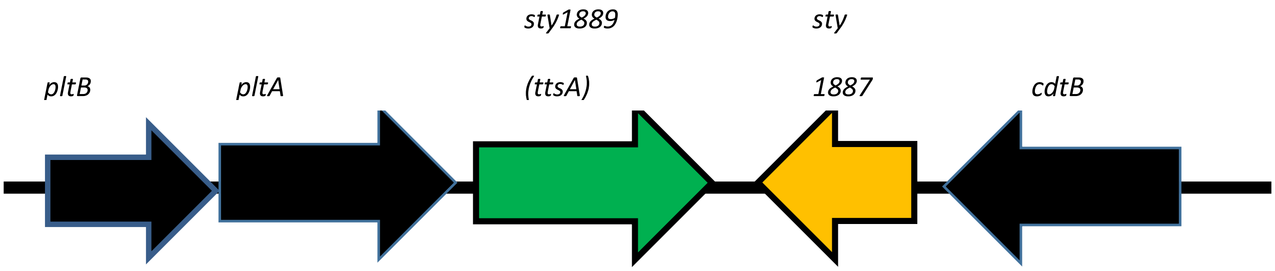

C

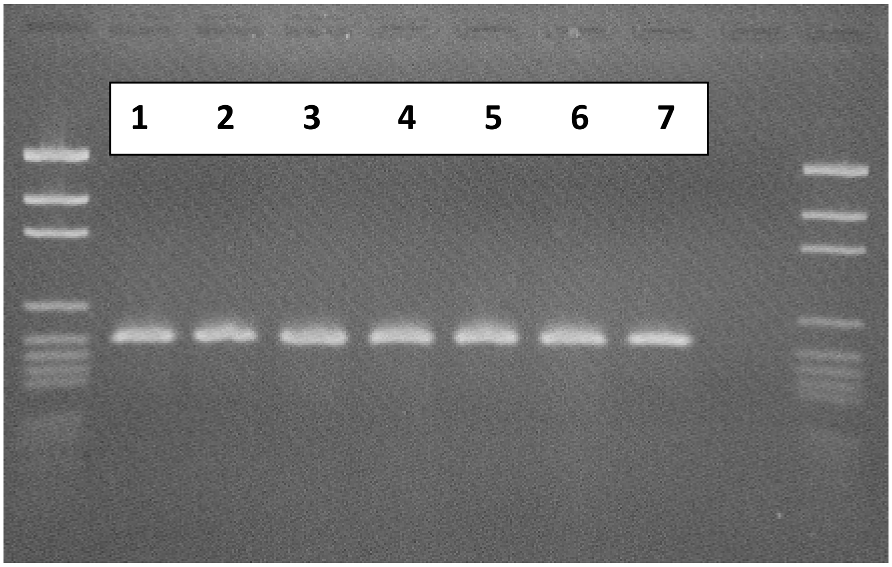

Additional File 4. (A) Sequence of *cdtB* and surrounding region, indicating that deletion is internal to *cdtB*; *cdtB* start and stop codons are boxed. Primer sequences in bold. Primer sequence homologous to pKD4 template primer P1 and P2 are italicized. 'Scar' sequence from pKD4 in orange. The sequence shown is for the serovar Javiana mutant; the serovar Schwarzengrund and Montevideo mutants showed equivalent sequences. (B) Organization of the region that contains *pltB*, *pltA*, and *cdtB*, which indicates that the *cdtB* deletion shown in (a) will not disrupt *pltA* or *pltB*. (C) Gel that shows the PCR product for the *cdtB* deletion alleles in serovars Schwarzengrund (lane 1), Montevideo (lane 4), and Javiana (lane 7); these PCR products were used to generate the sequencing data shown in part (A).
